# The Role of Sexual Selection and Conflict in Mediating Among-Population Variation in Mating Strategies and Sexually Dimorphic Traits in *Sepsis punctum*

**DOI:** 10.1371/journal.pone.0049511

**Published:** 2012-12-05

**Authors:** Caitlin Dmitriew, Wolf U. Blanckenhorn

**Affiliations:** 1 Department of Ecology and Evolutionary Biology, University of Colorado at Boulder, Boulder, Colorado, United States of America; 2 Institute of Evolutionary Biology and Environmental Studies, University of Zurich, Zurich, Switzerland; University of Melbourne, Australia

## Abstract

The black scavenger fly *Sepsis punctum* exhibits striking among-population variation in the direction and magnitude of sexual size dimorphism, modification to the male forelimb and pre-copulatory behaviour. In some populations, male-biased sexual size dimorphism is observed; in other, less dimorphic, populations males court prior to mating. Such variation in reproductive traits is of interest to evolutionary biologists because it has the potential to limit gene flow among populations, contributing to speciation. Here, we investigate whether large male body size and modified forefemur are associated with higher male mating success within populations, whether these traits are associated with higher mating success among populations, and if these traits carry viability costs that could constrain their response to sexual selection. Flies from five distinct populations were reared at high or low food, generating high and low quality males. The expression of body size, forelimb morphology and courtship rate were each greater at high food, but high food males experienced higher mating success or reduced latency to first copulation in only one of the populations. Among populations, overall mating success increased with the degree of male-bias in overall body size and forelimb modification, suggesting that these traits have evolved as a means of increasing male mating rate. The increased mating success observed in large-male populations raises the question of why variation in magnitude of dimorphism persists among populations. One reason may be that costs of producing a large size constrain the evolution of ever-larger males. We found no evidence that juvenile mortality under food stress was greater for large-male populations, but development time was considerably longer and may represent an important constraint in an ephemeral and competitive growth environment.

## Introduction

One of the classic problems in evolutionary biology is to identify the factors underlying the diversity of species on earth. To a large extent, speciation appears to be driven by the exploitation of ecological niches [1], but sexual selection is also thought to play an important role in initiating or reinforcing reproductive isolation among populations [2]–[6].

Theory suggests that coevolutionary change due to sexual conflict may be particularly rapid [7]. Sexual conflict is a consequence of the fundamental difference in male and female reproductive strategies, which generates a mismatch in the optimal mating rate for each sex. The optimal mating rate of males is typically much higher than that of females [8]–[10]. As a result, selection on traits that promote male persistence and female resistance to mating has been shown to result in an evolutionary arms race among species and populations of a species [11], [12]. Active female preference for signal traits such as male ornaments or courtship may also evolve rapidly through the chase-away process [10]. In this model, females evolve an ever-higher threshold of response to a male signal, selecting for a stronger male signal until a limit is imposed by the viability cost of producing the trait.

Rapid coevolution due to sexual selection may result in the amplification of small differences in the initial conditions, promoting differentiation among reproductively isolated populations [13]. These differences may arise as the result of random factors such as mutations in the genes controlling male phenotype (which might exploit a pre-existing female preference) [14], or by variation in local ecological conditions [13], [15]–[21]. For example, female preference for male colouration in guppies varies in response to water colour and predation regime, both of which can vary considerably over even limited spatial scales [22]–[23]. Whatever the underlying cause, as populations diverge in mating preferences or strategies, rates of gene flow upon secondary contact are expected to decline as mate recognition or the fitness of hybrid offspring declines [24]–[25]. However, the probability that reproductive isolation will ultimately result may depend upon which sex has greater control over mating, since the exertion of female mating preferences may play an important role in reinforcing reproductive isolation [1], [25].

The black scavenger fly *Sepsis punctum* (Diptera: Sepsidae) is an ideal system for the study of the role of sexual selection in promoting the divergence of populations. In this species, both mating behaviour and morphology differ dramatically among populations. We examined how female mating preferences may shape male trait evolution by evaluating the relationship between trait expression and male mating success within and among populations.

### The Study System

**Figure 1:**
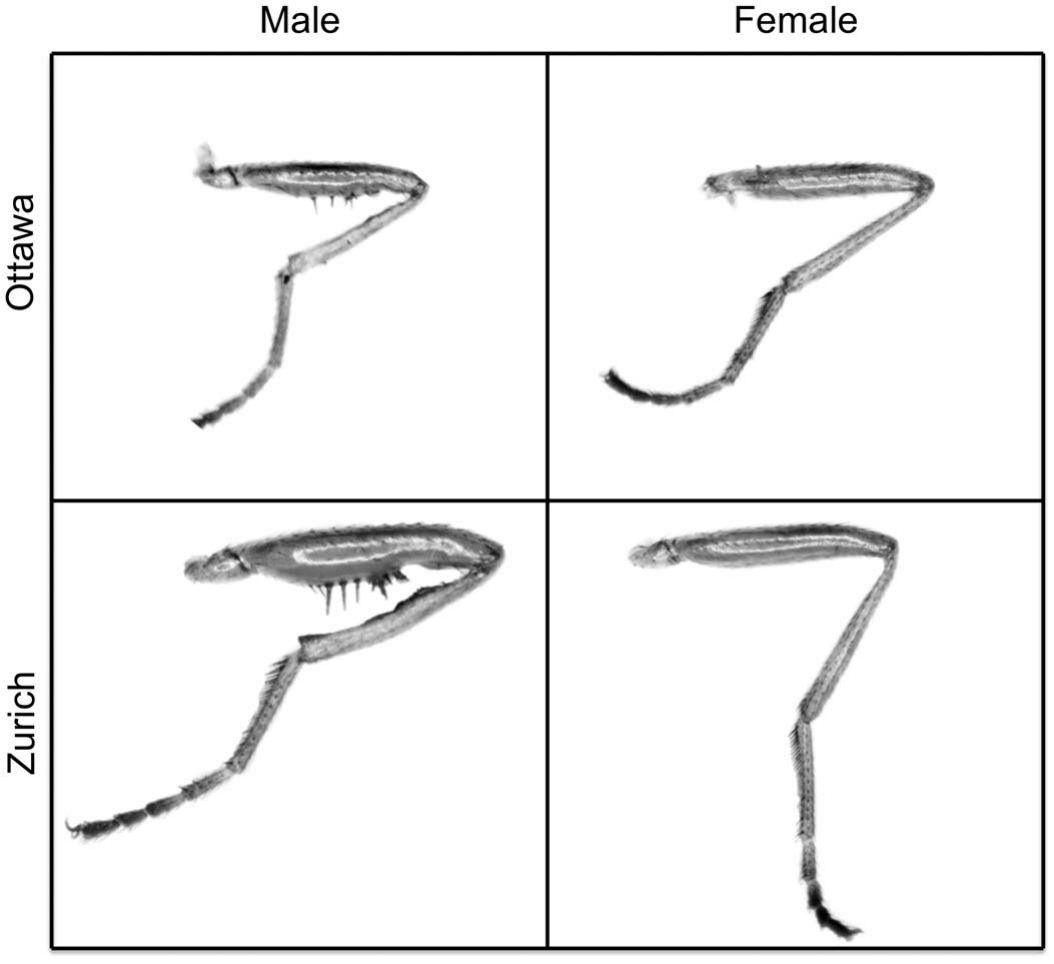
Sexual dimorphism of the forefemur in two populations of *S. punctum*.

*S. punctum* is a geographically widespread species occurring throughout North America and Europe and extending into Western Asia [26]. This coprophagous species develops in vertebrate excrement, primarily cow dung but also that of dogs, horses and other species. In *S. punctum* originating from North America, females are slightly larger than males, whereas in Europe males are typically much larger than females in terms of overall body size [27]. Both European and North American *S. punctum* males possess modified forelimbs including spines at the ventral margins of the forefemur, but the degree of forelimb elaboration is notably reduced in North America (Figure 1). Forelimb spines occur in 28 of the 31 sepsid species studied by Puniamoorthy *et al.* [28], and their modification matches precisely the use of forelimbs for grasping females at the wing base during copulation. Such morphological modifications have been shown to confer an advantage in pre-copulatory struggles with unwilling females in several insect species, independent of body size [29]–[31], including in the closely related species *Sepsis cynipsea*, for which costs of mating and resistance have been demonstrated [32]. Both large size and forelimb elaboration are presumed to increase the male’s ability to grasp reluctant females during copulation. Even though sexual size dimorphism is typically female biased in insects, large males often have an advantage in intra-sexual contests and are better at overcoming female resistance to mating [16], [33]–[37]. We hypothesize that male mating success varies in a population-specific way, either through their function as a signal that appeals to choosy females or by increasing the male’s ability to grasp females during copulation.

European and North American *S. punctum* also differ in pre-mating behaviour. In Europe, scramble competition for access to mates predominates; only in North American populations do males court females by facing them and vigorously waving their abdomen from side to side prior to mounting attempts [38]. This pre-copulatory courtship, which has not been observed in any European populations of *S. punctum,* is hypothesized to act as a signal of genetic quality or condition to a prospective mate, leading to our prediction that large, high-condition males (reared at abundant food) will express courtship at a higher rate and experience greater mating success than small, low-condition males. Despite all these differences, cross-continental populations (including those used in this study) readily hybridize, though no data on the relative fitness of hybrid offspring are available (W.U. Blanckenhorn et al., unpublished data, [39]).

The reasons for the observed among-population variation in male phenotype are at present unknown, though sexual conflict over mating rate is hypothesized to be a contributing factor. The apparent diversification of mating strategies may be further influenced by other ecological factors that vary among the populations of study, or by genetic drift within isolated populations. We focus on the costs and benefits of producing a large overall body size, elaborated forelimb and courtship behaviour. While large body size is generally associated with higher fitness in both males and females [40]–[42], at some point the costs of producing or maintaining a large body will exceed the advantages. The optimal size, which balances the costs and benefits of being big, may vary depending upon local ecological conditions and selective pressures. Whether driven by ecological variation or by sexual conflict, the behavioural and morphological traits described in this system have the potential to restrict gene flow among populations, ultimately leading to speciation. For example, if a trait preferred by females of one population is absent in the males of another population, mating may be suppressed. Females of different populations may also be more or less reluctant to mate, meaning that insufficiently large males will experience reduced mating success in hybrid pairs.

In this study, we attempt to relate within- and among-population variation in male secondary sexual traits to success in mating by comparing mating success of high and low quality males and males originating from different populations. We address two main questions. First, do the traits in question in fact increase mating success within and among populations? Second, might sexual selection for large male size or exaggerated forelimbs be countered by a reduction in juvenile viability under food stress, or by a substantial prolongation of development time? We begin by assessing the relationship between male condition, phenotype, and reproductive success by rearing males from each of five geographically distinct populations at high or low food. Low food males are predicted to have a lower mating success in all populations, either because they are less able to overcome female resistance to mating or are less effective at courting [16]. We then compared mating success among populations with different mean trait values with the expectation that overall mating success would increase with male body size and forelimb width. Lastly, we addressed the possibility that the reproductive advantages of a large male body size trade off against viability, by comparing the effect of food stress on juvenile survival and development time among populations varying in male body size.

## Methods

### General

Flies were collected in the spring and summer of 2010 from three sites in Europe and two in North America: Zurich, Switzerland (Z) 47°21′N 8°31′E; Berlin, Germany (B) 52°30′N 13°25′E; Lake Trasimeno, Italy (I) 43°08′N 12°06′E; Ottawa, Ontario (O) 45°25′N 75°41′W; Park City, Utah (PC) 40°39′N 111°29′W. Flies were obtained by placing multiple containers of cow dung in the field for 24 hours to allow oviposition by *S. punctum* females. No special permits were required for sampling this species, and collection sites were on public land. These containers were shipped to the lab and placed inside large plastic bottles capped with nylon mesh until eclosion of the offspring. *S. punctum* adults eclosing from each bottle were conservatively assumed to be the progeny of a single female; multiple females are sometimes observed on the artificial dung pat (C. Dmitriew, personal observation). They were maintained thereafter in isofemale lines with fresh dung added every 3–4 weeks. Populations were comprised of 11–24 lines. Lines were housed in 1 L plastic bottles at 19°C, and provided with sugar and water *ad libitum*.

Due to the large number of populations, observations were staggered (Ottawa and Zurich beginning November 4, 2010; Park City on November 18, Berlin on Dec. 4, and Italy on Feb. 6, 2011). All experimental trials were conducted using identical conditions (described below) in the same environmental chamber. The dung used for rearing the flies from all populations was from the same batch of cattle dung (collected in the vicinity of Zurich in July 2010, thoroughly homogenized and frozen in 1 L containers at −80°C).

### Food Manipulation

An identical protocol was used for all populations. Male and female flies were randomly paired in glass vials. At 9∶00 h, oviposition substrate (cattle dung) was introduced into the vial and removed after 6 hours. Because of the fragility of the eggs, larvae were transferred after hatching to experimental conditions 24 hours later. Thus, all larvae were introduced into the treatment conditions within a 6–8 hour window.

Test populations were comprised of 81–243 individuals per treatment, with the exception of Z and O, which formed part of a second, larger scale experiment (C. Dmitriew, in prep.). For these two populations, a randomly selected subset of adult flies (N = 200 per treatment) was used for the assessment of morphological traits. Values for juvenile viability were calculated using the entire larval data set (N = 1468 and N = 1744 for Z and O, respectively).

In the high food (H) treatment, larvae were reared individually in 3 ml plastic containers filled level with *ca.* 3.5 g homogenized dung. This represents a large excess of dung for the development of a single larva in this species (Dmitriew, pers. obs.). In the low food (L) treatment, dung was diluted (50% by mass) with agar (2 g agar/100 ml tap water) and thoroughly homogenized. The caps of 1.5 ml centrifuge tubes were filled level with this solution (approx. 0.3 g), an amount of dung that was found to produce a substantially small body size in pilot studies. A single larva was placed in each container, which was in turn kept in a 60 mm plastic Petri dish. Larvae in both treatments were reared to eclosion in an environmental cabinet with a 16∶8 L:D photoperiod at 21.5°C.

### Effect of Population and Food Treatment on Morphology, Development, and Juvenile Survivorship

Following mate trials, experimental flies were freeze-killed and stored individually in centrifuge tubes at −20°C until dissection. The right foreleg, midleg, hindleg and wing (unless clipped for mate trials in which case the left wing was used) were glued flat and photographed under a microscope (Leica Firecam V. 3.4.1 © Leica Microsystems). The right side of the thorax was photographed separately. A total of seven measures were recorded using the program tpsDIG2 (© F. J. Rohlf 2010; http://life.bio.sunysb.edu/morph/soft-dataacq.html) and converted to linear measurements using the program PAST (© O. Hammer 2003; http://folk.uio.no/ohammer/past/index.html). These measures were forefemur length and width, forefemur length, midtibia length, hindtibia length, thorax length, and wing length (measured along the R_4–5_ vein from the r-m crossvein to the wingtip).

We conducted a principal components analysis on the above seven traits to identify the most appropriate measure of overall body size. The first and second eigenvectors accounted for 78.3% and 11.2% of the variation, respectively, when all data of both sexes were pooled across populations (Table 1). The first eigenvector corresponds to overall body size, with loadings of all traits being high and positive. We chose hindtibia length as our proxy for overall body size; this trait loaded heavily onto PC1 in both sexes and was used to standardize forefemur width to overall body size. Effect of population, population nested within continent and food treatment on adult size, growth rate and development time were assessed using ANOVA. In order to obtain a relative measure of forefemur width (FFW), we used ANCOVA with hindtibia length as the covariate.

**Table 1:** Results of principle components analysis for seven metrics of body size (THO = thorax, FFL = forefemur length; FFW = forefemur width; FTL = foretibia length; MTL = midtibia length; HTL = hindtibia length; WL = wing length).

		Eigenvector						
PC	Eigenvalue	Thorax	FFL	FFW	FTL	MTL	HTL	WL
1	5.480	0.405	0.408	0.324	0.396	0.414	0.399	0.279
2	0.790	−0.023	−0.061	−0.573	0.160	−0.007	−0.151	0.787

Containers were checked for adult eclosion daily, and flies were sexed within 24 hours of eclosion. Development time was recorded as the length of time in days between egg laying and eclosion. Juvenile survival was assessed as the % of experimental larvae that eclosed as adults. Sex ratio was calculated from each population by treatment combination as the % of eclosing adults that are male.

### Effect of Population of Origin and Food Manipulation on Mating Behaviour

Males were briefly anaesthetized with CO_2_ and the tip of either the left or right wing of each male was clipped for identification (leaving them capable of flight). Virgin males and females were placed individually in glass vials (25 mm in diameter and 10 cm in length) with sugar *ad libitum* and cotton soaked in water. 24 hours prior to mating trials, females were provided with dung to stimulate egg production and receptivity to mating. For each trial, a single female (always from the high food treatment) was placed in a vial with dung and two males randomly selected from the same rearing treatment (either high or low food). Pairs of males were used to increase the likelihood of mating; at these relatively low densities, male-male interactions were rare [39].

Groups were observed for a two-hour period. We noted whether a copulation occurred in a given mate trial and also the latency to first copulation, which we considered to be a measure of the female’s reluctance to mate. We counted only the first copulation in each trial; remating within the two-hour period was rare; thus, mating success is calculated for each population and treatment combination as the number of mating trials that resulted in at least one copulation divided by the total number of trials conducted. Latency to first copulation was recorded as a secondary measure of reluctance to mate. The presence or absence of pre-copulatory courtship behaviour was also noted for each individual.

## Results

### Effect of Population of Origin and Food Treatment on Morphology

**Figure 2:**
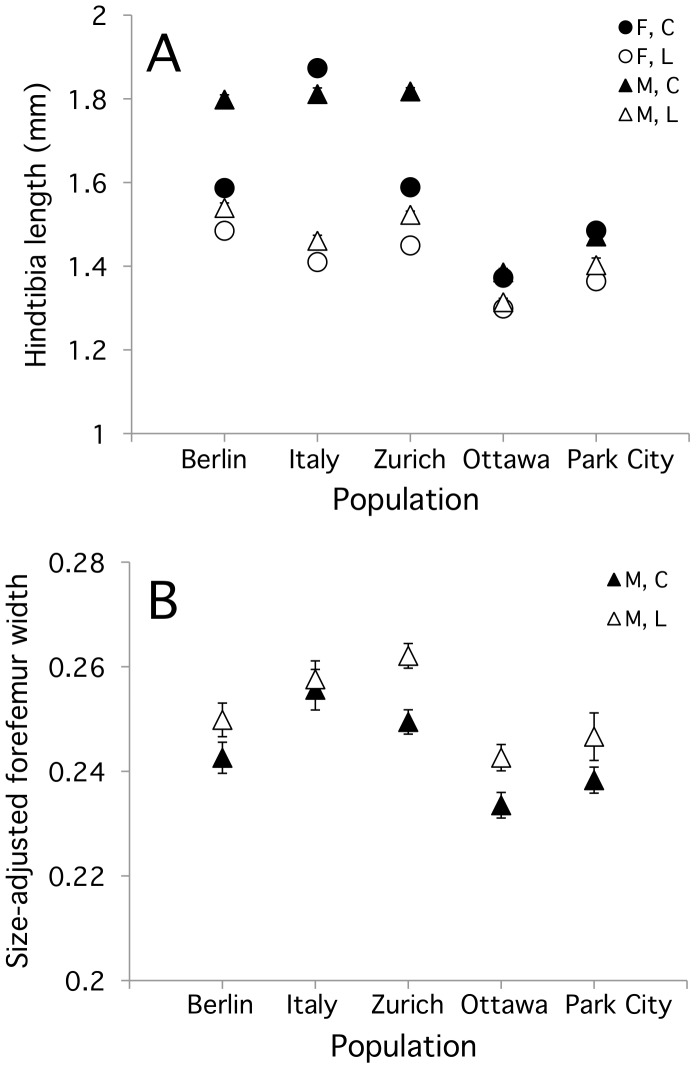
Effect of food treatment and sex on (A) hindtibia length and (B) size-adjusted forefemur width (see text) in five populations of *S. punctum* originating from Europe and North America. Filled symbols represent the high food treatment and open circles low food, while circles and triangles are males and females, respectively. Error bars represent +/−1 SE from the mean, and when not visible are smaller than the symbols.

In all three European populations, males were larger than females, with the exception of the Italy high food treatment group, in which males and females had similar HTL (Tukey’s Posthoc Test: M = F). The pattern of sexual size dimorphism was different in the North American populations, with all population by treatment combinations being sexually monomorphic (Figure 2*a*). While male-biased sexual size dimorphism was observed in both treatments in Europe, it was less pronounced under food stress because the food manipulation had a stronger negative effect on male size than on female size (Figure 2*a*, significant food treatment by sex (by population) interaction in Table 2).

**Table 2:** Effect of continent and population of origin and larval food treatment on (a) hindtibia lenth, (b) relative male forefemur width, (c) male development time in days, (d) male growth rate (growth of hindtibia length in mm/day).

	Source	d.f.^1^	*F*	*P*
*(a) Hindtibia length*	Trt	1	1210.16	<.0001
	Continent	1	1982.87	<.0001
	Pop(Continent)	3	54.55	<.0001
	Sex	1	110.09	<.0001
	Continent*Trt	1	332.79	<.0001
	Trt*Pop(Continent)	3	62.68	<.0001
	Trt*Sex	1	3.84	0.050
	Continent*Sex	1	62.21	<.0001
	Sex*Pop(Continent)	3	33.35	<.0001
	Trt*Continent*Sex	1	21.59	<.0001
	Trt*Sex*Pop(Continent)	3	27.22	<.0001
	Error	19		
	Total	1471		
*(b) Relative forefemur*	Trt	1	1.45	0.23
*width (males)*	Continent	1	31.62	<.0001
	Pop(Continent)	3	2.59	0.052
	HTL	1	109.78	<.0001
	Continent*Trt	1	8.52	0.0036
	Trt*Pop(Continent)	3	0.53	0.66
	Trt*HTL	1	7.83	0.0053
	Continent*HTL	1	1.35	0.25
	HTL*Pop(Continent)	3	1.98	0.12
	Trt*Continent*HTL	1	3.39	0.07
	Trt*HTL*Pop(Continent)	3	1.24	0.29
	Error	19		
	Total	699		
*(c) Development time*	Trt	1	81.49	<.0001
*(males)*	Continent	1	428.93	<.0001
	Pop(Continent)	3	15.37	<.0001
	Continent*Trt	1	11.15	0.0009
	Trt*Pop(Continent)	3	2.71	0.044
	Error	9		
	Total	704		
*(d) Growth rate*	Trt	1	139.88	<.0001
*(change in HTL over*	Continent	1	127.15	<.0001
*development time)*	Pop(Continent)	3	12.92	<.0001
*(males)*	Continent*Trt	1	72.73	<.0001
	Trt*Pop(Continent)	3	1.94	0.12
	Error	9		
	Total	700		

1 Reduced DF for some measures due to damage.

**Figure 3:**
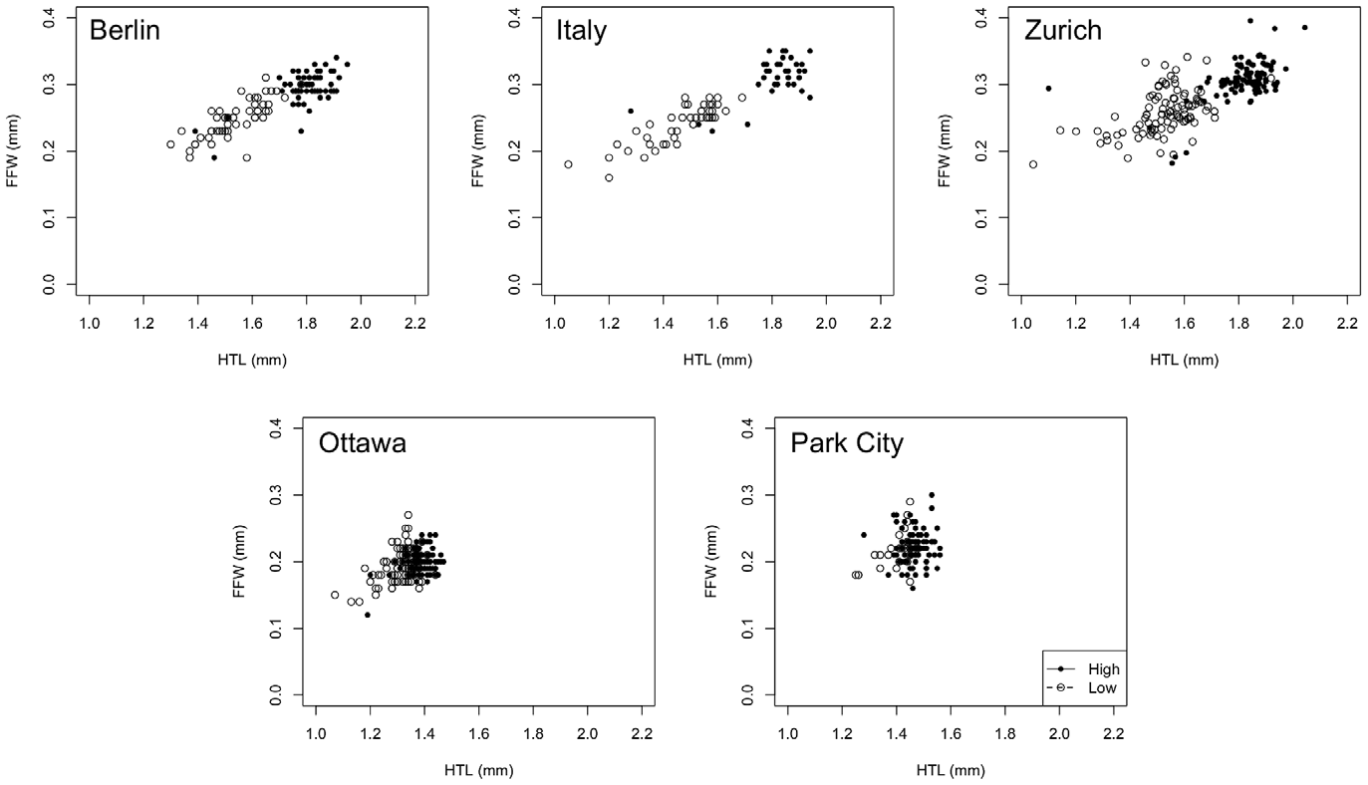
Regression of individual forefemur width on hindtibia length for males reared at high (solid symbols) and low (open symbols) larval food.

Size-adjusted forelimb width was smaller in the North American populations (PC and O) than in European populations (Figure 2*b*). Interestingly, FFW relative to body size tended to be greater at low food (interaction between food treatment and the covariate HTL, Table 2*b*, Figure 2), though in absolute terms the forefemur was reduced in size relative to the high food treatment in all populations (Figure 3; Continent: F_1,694_ = 55.1, *P* = 0.005; Treatment: *F*_1,694_ = 46.9, *P* = 0.006; Continent*Treatment: F_1,694_ = 30.0, *P* = 0.011; Population(Continent): *F*_3,694_ = 4.2, *P = *0.13; Treatment*Population(Continent) F_3,694_ = 3.9, *P*<0.009).

### Growth Rate, Development Time and Juvenile Survival

Development time was longer, and growth rate higher, in European compared to North American males (Table 2*cd*, 3). Low food caused a reduction in development times in all populations, to varying degrees, but growth rate was significantly reduced only in the European males (Table 3; Tukey’s *post hoc*: Berlin: H>L, Italy: H>L, Zurich: H>L, Ottawa: H = L, Park City H = L).

**Table 3:** Development time and growth rate of males by treatment and population of origin.

		Development time		Growth rate	
		Days (SE)		HTL/development time (SE)	
Continent	Population	High food	Low food	High food	Low food
Europe	Berlin	12.6(0.09)	12.1(0.09)*	0.143(0.001)	0.127(0.001)*
	Italy	13.2(0.11)	12.5(0.1)*	0.137(0.002)	0.117(0.001)*
	Zurich	13.1(0.07)	12.2(0.07)*	0.139(0.001)	0.125(0.001)*
N. America	Ottawa	11.4 (0.07)	11.0(0.07)*	0.122(0.001)	0.120(0.001)
	Park City	11.8 (0.07)	11.5(0.13)*	0.125(0.001)	0.122(0.002)

* Significant effect of treatment according to Tukey’s Post-hoc Test.

Juvenile viability was not significantly affected by larval growth treatment, with the exception of the monomorphic Ottawa population, for which survival was significantly lower in the low food treatment (Table 4). We also tested the prediction that the sex ratio would be female biased, particularly in the European populations. We found no consistent patterns (Table 4): the sex ratio was female-biased in the low food treatment for one population from each continent (Zurich and Park City), and in one population (Berlin) the sex ratio was unexpectedly and significantly male-biased. In the remaining populations, Ottawa and Italy, we saw no significant deviation from a 1∶1 sex ratio.

**Table 4:** Effect of food treatment and population of origin on juvenile viability and sex ratio at eclosion.

	Juvenile viability			Sex ratio at eclosion		
	probability of survival			M:F		
Population	High food	Low food	X^2^, df	High food	Low food	X^2^, df
Berlin	0.92	0.83	3.8, 1	51∶64	54∶39	3.9, 1*
Italy	0.86	0.86	0.0, 1	55∶38	41∶46	3.4, 1
Zurich	0.89	0.91	1.1, 1	240∶241	222∶280	6.7, 1**
Ottawa	0.95	0.89	15, 1 ***	221∶222	205∶217	0.34, 1
Park City	0.81	0.76	0.4, 1	86∶98	27∶42	4.0, 1*

* *P*<0.05;

** *P*<0.01;

*** *P*<0.001.

### Mating Behaviour

Although mating success varied strongly among populations and between continents, we found that the overall effect of food treatment was marginal but not significant (Logistic model for mated v. unmated pairs: Continent: d.f. = 1, X^2^ = 112.6, *P*<0.0001; Treatment: d.f. = 1, X^2^ = 3.02, *P*<0.08; Continent*Treatment: d.f. = 1, X^2^ = 1.38, *P* = 0.24; Population(Continent): d.f. = 3, X^2^ = 19.8, *P* = 0.0002; Treatment*Population(Continent) d.f. = 3, X^2^ = 0.90, *P* = 0.83. We conducted post-hoc Chi-square tests for individual populations, which showed that low food males mated significantly less often in the Zurich population (Table 5), for which the sample size was considerably larger. Low food males from this population also tended to have a much longer latency to first copulation, as reflected in the interaction between food treatment and population on latency to mating (Continent: F_1,190_ = 0.0041, *P* = 0.95; Treatment: *F*_1,190_ = 0.28, *P* = 0.60; Continent*Treatment: F_1,190_ = 0.72; Population(Continent): *F*_2,190_ = 14.2, *P*<0.0001; Treatment*Population(Continent) F_2,190_ = 6.04, *P*<0.003; Table 5). Ottawa is not included in the analysis due to the very small number of matings observed.

**Table 5:** Behavioural differentiation in copulation and courtship frequency, and latency to first copulation by population and treatment.

	% copulations observed			% individuals courting			Latency to first copulation	
	(# pairs)			(# individuals)			minutes (SE)	
Population	High food	Low food	X^2^, df	High food	Low food	X^2^, df	High food	Low food
Berlin	0.79 (24)	0.88 (24)	0.60, 1	0.0 (42)	0.0 (52)		35.1 (10.7)	25.9 (10.2)
Italy	0.95 (16)	0.94 (20)	0.03, 1	0.0 (40)	0.0 (16)		10.4 (12.1)	22.3 (10.7)
Zurich	0.79 (90)	0.63 (51)	4.31, 1 *	0.0 (106)	0.0 (198)		36.6 (5.7) *	84.1 (6.9)
Ottawa	0.05 (20)	0.0 (18)	0.92, 1	0.85 (40)	0.59 (36)	6.01, 1 *	.	.
Park City	0.38 (21)	0.13 (16)	2.67, 1	0.38 (42)	0.14 (28)	4.673, 1 *	35.3 (16.5)	38.6 (33.0)

* *P*<0.05 Tukey’s Post-hoc test.

Courtship was observed in both North American populations, with the Ottawa flies courting at a much higher rate than the Park City flies (Table 5). In both populations courtship frequency was greater at high food (Table 5), though low mating success in both populations precluded the calculation of a relationship between courtships and mating success. As expected, no courtship behaviour was observed in the European populations.

### Relationship between Male Phenotype and Mating Success Among Populations

**Figure 4:**
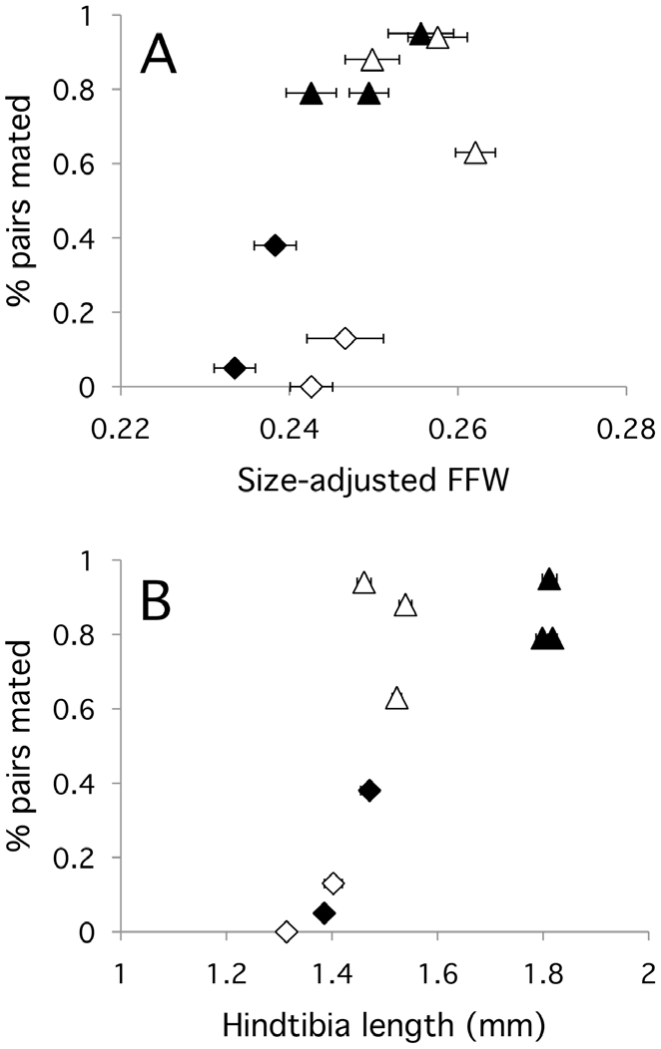
Relationship between probability of mating in a given mating trial and population mean (A) male hindtibia length and (B) size-adjusted forefemur width. European populations are represented with triangles, and North American populations with diamonds. Filled and open symbols represent high and low food treatments, respectively. Error bars show +/−1 SE from the mean.

Comparing mating frequency (the percentage of trials in which a copulation was observed) across populations, we found that mating success increased with population mean male body size (Figure 4). Although males were smaller at low food, the relationship between mating frequency and size was similar in both treatments (R^2^ = 0.81; HTL: F_1,9_ = 21.8, *P* = 0.0034; Treatment: F_1,9_ = 8.06, *P* = 0.030; HTL*Treatment: F_1,9_ = 3.43, *P* = 0.11). The same pattern was observed for the males’ size-adjusted forefemur width (R^2^ = 0.64; relative FFW: F_1,9_ = 10.5, *P* = 0.018; Treatment: F_1,9_ = 3.57, *P* = 0.11; relative FFW*Treatment: F_1,9_ = 0.0003, *P* = 0.99).

## Discussion

Our results demonstrate that the considerable variation in male body size and forefemur elaboration observed among populations of *S. punctum* corresponds tightly to variation in population-mean mating success. In populations where males were similar to or smaller in size than females, mating success was very low, while in populations having the largest males, mating success was much higher. Both theory and empirical work suggest that the optimal mating rate for females is lower than that of males, causing mating rate to be an important target of sexual conflict [43]. This is because while male fitness typically increases with each copulation, females gain diminishing returns from multiple matings [8], [43]. Our data support the hypothesis that both male-biased sexual size dimorphism (atypical among insects) and an elaborated forefemur each function independently to increase mating success, thereby allowing males to achieve higher fitness. Another possibility is that females from large male populations have a higher optimal mating rate, perhaps because the costs of resisting a large male are greater than the costs of accepting additional matings (e.g. [44]) or because the benefits of polyandry vary among populations. These effects could be unraveled by assessing the costs female *S. punctum* incur by mating relative to the costs they incur for resisting male mating attempts, and whether these vary among populations.

A previous study [27] found strong positive sexual selection on male body size in four European *S. punctum* populations (including Zurich and Berlin) and weak positive selection on three North American populations. In this study, competing males were of a wide range of sizes, produced by rearing flies at high or low densities. These data suggest that body size is under sexual selection. However, whether relative forefemur width is positively associated with male mating success within populations remains to be demonstrated by comparing mating success of males with relatively large and relatively small forefemurs. In some species, the effect of morphological adaptations that aid in overcoming female resistance may depend on the relative body size of the male and female, as has been observed in water striders [12]. Such an effect could explain the finding that forefemur width was larger relative to body size at low food. Males in poor overall condition, such as the flies in our low food treatment group, may compensate to some extent by maintaining investment in forefemur even when they are unable to reach a larger body size.

Contrary to expectation, mating success was not higher for high food males, with the exception of one population (Zurich), which also mated sooner than low food males. This suggests that in this population, females were either less likely to resist mating with large males, or large males were better able to overcome female resistance to mating. However, mating success was similar for both treatments in the other four populations. We note that the use of virgin females, which may be less selective than females that experience multiple mating opportunities, could have caused us to underestimate the importance of body size in determining mating success. The fact that we had a much larger sample size for Zurich than for the other four populations suggests that we had limited power for detecting an effect of food treatment on mating behaviour.

### Does Sexual Conflict Over Mating Rate Contribute to Among-population Variation in Mating Traits?

Our results suggest a strong relationship between male size, forelimb elaboration and male mating success among populations. In arthropods, female-biased sexual size dimorphism is the rule [45], presumably because fecundity selection on female body size tends to be stronger than sexual selection on male body size [41], [46]. Exceptional cases of male-biased sexual size dimorphism in insects, such as in European *S. punctum*, can arise by three mechanisms: either as a consequence of male-male competition for access to females, by female choice of large males, or because large males are better able to overcome female reluctance to mate [16], [34], [47]. We did not observe many direct interactions among males mediating access to females in our experiment, and this was the same in Puniamoorthy et al. [27]. It therefore seems more probable that larger males achieve higher mating success as the result of either active or passive female preferences [16], [34]. In addition to the apparent advantage of a large male size, comparative evidence across the Sepsidae clearly demonstrates that male forelimb spines function in grasping the female wingbase [28], [48]. Whether their evolution is attributable to selection for male persistence in mating indicating sexual conflict is debated, however [16], [48]. Female *S. punctum* are frequently observed to resist copulation by vigorous shaking (C. Dmitriew pers. obs.), a behaviour that is also common in the better-studied congener *S. cynipsea* [16], [32]. Unfortunately, we did not collect data on this behaviour in the current study, and cannot say whether this behaviour varies significantly among populations. In *species. cynipsea*, females housed with a male had reduced survivorship than those housed with another female [32]. These observations are strongly suggestive of a mismatch between male and female optimal mating rates in this group, and could select for larger male body size and forelimb exaggeration in males. A result of this possible example of conflict could be an arms race between males and females that is at different stages of escalation in different populations [11], [12], [49] Female *S. punctum* have no obvious morphological adaptations conferring such resistance, suggesting that females exert control over mating primarily through behavioural adaptations though a larger female body size (such as that as observed in European females relative to N. American females) could also aid in female resistance as well. A final possibility is that differentiation among populations in mating rate is driven by ecological variation. For example, differences in predation regimes, resource availability or sex ratio can all influence the optimal mating rate of females [17], [44], [50].

Further studies are required to establish whether there is a cost to mating (or resistance to mating) in females [cf. 32, 51], and whether females from those populations with the strongest male size-bias are better able to resist copulations, as predicted under the model of sexually antagonistic coevolution [9], [11].

### Costs of Large Male Body Size

We found no support for our hypothesis that European populations with large males would be more susceptible to food restriction during development, thereby constraining the evolution of ever-larger body size and perhaps explaining the persistence of small-male populations despite the apparent advantages in mating success. However, large male populations had longer average development times and grew faster, both of which could result in important costs for these males. Given the benefits associated with large body size for both sexes in a wide range of taxa [33], [52], it is assumed that costs of producing and/or maintaining a large body limit evolution towards larger size [46]. These costs may include costs of growth itself, such as the risk of predation upon active feeders, long-term costs associated with oxidative damage or reduced energy storage [4], [53], or costs associated with prolonged development time [46]. Both growth acceleration and prolonged development time were observed in our large-male populations of *S. punctum*. While our study cannot ascribe specific fitness costs to these effects, they merit further investigation, particular considering the ecology of the species. Dung pats are highly competitive, ephemeral environments: both conspecifics and a number of other dung-feeding species compete for this ephemeral resource, and there are various predators and parasitoids associated with the dung environment (reviewed in [54]). Future studies might productively investigate the potential trade-of between large body size and long- and short-term fitness costs due to rapid growth or prolonged development time [55]. Differences in competition or predation regimes, or in the rate of dung pat drying, likely exist among populations and continents, influencing the relative risks and contributing to the variation in behaviour and morphology observed among populations.

### Potential for Speciation

The nature of the trait variation among populations of *S. punctum* could contribute to the evolution of reproductive isolation. Although hybridization between European and North American flies results in the production of viable offspring [38], the differences in reproductive traits among populations could reduce the reproductive success of hybrids. This system could therefore serve as a model to test the prediction that the evolution of reproductive isolation between two populations depends upon which sex has greater control over mating rate [25]. According to this model, female control over mating should reduce hybridization because they are better able to exercise mating preferences; females are likely to resist mating with males whose signal traits or body size are outside of a preferred range. As males are expected to be less discriminating due to the relatively low cost of mating, hybridization is more likely to occur when males exert greater control over mating rate within a population. Under this model, the rate of speciation in *S. punctum* may be accelerated in North American relative to European populations.

### Summary

In conclusion, our experiment provides first evidence regarding the role of sexual selection in shaping among-population variation in *Sepsis punctum*. Male body size, forelimb width and courtship varied considerably by population of origin, and across populations, the former two traits were positively associated with mating rate. Given the many advantages of large body size [43], the restriction of the large-male phenotype to European populations is puzzling. Costs of prolonged development time associated with large size as well as the enhanced female control of the mating outcome in North American populations with male pre-copulatory courtship are hypothesized to contribute. It is currently unclear which mating system is ancestral, but molecular phylogenetic studies are planned to resolve this issue. In either case, the existing differences could eventually lead to complete reproductive isolation among populations, particularly in populations in which females exert greater control over mating.
